# Molecular mechanisms of cisplatin-induced nephrotoxicity: a balance on the knife edge between renoprotection and tumor toxicity

**DOI:** 10.1186/s12929-019-0518-9

**Published:** 2019-03-13

**Authors:** Vladislav Volarevic, Bojana Djokovic, Marina Gazdic Jankovic, C. Randall Harrell, Crissy Fellabaum, Valentin Djonov, Nebojsa Arsenijevic

**Affiliations:** 1 Center for Molecular Medicine and Stem Cell Research, Faculty of Medical Sciences, University of Kragujevac, 69 Svetozar Markovic Street, Kragujevac, 34000 Serbia; 2 Department of Genetics, Faculty of Medical Sciences, University of Kragujevac, Kragujevac, Serbia; 3 Regenerative Processing Plant, LLC, US Highway 19 N Palm Harbor, Palm Harbor, Florida, 34176 USA; 4 Institute of Anatomy, University of Bern, 2 Baltzerstrasse, Bern, Switzerland

**Keywords:** Cisplatin, Nephrotoxicity, Acute kidney injury, Apoptosis, Inflammation

## Abstract

**Background:**

Cisplatin (cis-diamminedichloroplatinum II, CDDP) is one of the most effective chemotherapeutic agents. However, its clinical use is limited due to the severe side effects, including nephrotoxicity and acute kidney injury (AKI) which develop due to renal accumulation and biotransformation of CDDP. The alleviation or prevention of CDDP-caused nephrotoxicity is currently accomplished by hydration, magnesium supplementation or mannitol-induced forced diuresis which is considered for high-dose CDDP-treated patients. However, mannitol treatment causes over-diuresis and consequent dehydration in CDDP-treated patients, indicating an urgent need for the clinical use of safe and efficacious renoprotective drug as an additive therapy for high dose CDDP-treated patients.

**Main body:**

In this review article we describe in detail signaling pathways involved in CDDP-induced apoptosis of renal tubular cells, oxidative stress and inflammatory response in injured kidneys in order to pave the way for the design of new therapeutic approaches that can minimize CDDP-induced nephrotoxicity. Most of these molecular pathways are, at the same time, crucially involved in cytotoxic activity of CDDP against tumor cells and potential alterations in their function might mitigate CDDP-induced anti-tumor effects.

**Conclusion:**

Despite the fact that many molecules were designated as potential therapeutic targets for renoprotection against CDDP, modulation of CDDP-induced nephrotoxicity still represents a balance on the knife edge between renoprotection and tumor toxicity.

## Background

Cisplatin (cis-diamminedichloroplatinum II, CDDP) is one of the most effective chemotherapeutic agents, widely used for the treatment of several malignant diseases including head and neck [1, 2], esophageal [3], bladder [4], testicular [5], ovarian [6], uterine [7], cervical [8], breast [9], stomach [10], non-small [11], and small-cell lung cancers [12]. CDDP crosslinks purine bases within DNA and interferes with DNA synthesis [13]. An impaired cell division is the main CDDP-based effect and, accordingly, CDDP shows highest activity in rapidly proliferating cells [13]. Therefore, CDDP-induced mucosal injury in gastrointestinal tract as well as myelosuppression due to the CDDP-caused injury of bone marrow, are severe and life-threatening side effects of CDDP-based therapy [14–17]. However, the most usually observed, dose-dependent and cumulative CDDP-caused side effect, noticed in 30–40% of patients, is nephrotoxicity [18–22]. CDDP-induced nephrotoxicity is manifested as acute kidney injury (AKI), salt or magnesium wasting and loss of urinary concentrating ability [18–22]. CDDP-caused renal dysfunction happens as a result of CDDP accumulation and biotransformation in the kidneys [18–22]. The alleviation or prevention of CDDP-caused nephrotoxicity is currently accomplished by short-duration and lower-volume hydration, magnesium supplementation (8–16 milliequivalents) or by mannitol-induced forced diuresis which is considered for high-dose CDDP-treated patients and/or patients with preexisting hypertension [23]. However, mannitol treatment causes over-diuresis and consequent dehydration in CDDP-treated patients, indicating an urgent need for the clinical application of safe and efficacious renoprotective drug, as an additive therapy for high dose CDDP-treated patients [24]. Until now, amifostine [(ethanethiol, 2-[(3-aminopropyl)amino] dihydrogen phosphate ester)] was the most commonly tested as nephroprotective agent against CDDP, but several serious side effects, including ototoxicity, hypotension, vertigo, hypocalciemia, severe nausea and vomiting, limited its clinical use [25, 26]. Although some of the other thiol-generating cytoprotective agents (sodium thiosulfate, reduced glutathione and diethyldithiocarbamate) appeared to reduce CDDP-caused nephrotoxicity, all of them have demonstrated an unwanted tumor protecting effect which restricted their clinical use [27, 28]. Consequently, there still remains an unmet need for the development of new, renoprotoctive agents in which activity should be relied on the modulation of pharmacokinetics and biological effects of CDDP in the kidneys. In this review paper, we emphasized current knowledge regarding molecular and cellular mechanisms involved in renal uptake, biotransformation and toxicity of CDDP in order to pave the way for new therapeutic approaches that can inhibit or minimize CDDP-dependent nephrotoxicity.

### Molecular mechanisms involved in renal uptake and accumulation of CDDP

During glomerular filtration and tubular secretion, CDDP accumulates in the kidneys [20]. Renal proximal tubular epithelial cells (PTECs) absorb molecules from primary urine and are mainly exposed to urinary excreted xenobiotics [29]. Accordingly, CDDP concentration in PTECs is about five times greater than in the blood [20]. Even non-toxic serum concentrations of CDDP may reach toxic levels in the kidneys, resulting in the development of renal dysfunction due to the severe injury of S3 segment of proximal tubules [30, 31].

An important process mediating cellular accumulation of CDDP is transporter-mediated uptake of this drug. Recent public data identified several different membrane transporters capable of transporting CDDP across the plasma membrane and across PTECs: the organic cation transporter 2 (OCT2), the copper transporter 1 (Ctr1) and the multidrug extrusion transporter 1 (MATE1) [32]. Among them, OCT2 is most important for renal uptake of CDDP while MATE 1 is mainly responsible for CDDP transportation from the proximal tubule to the urine [22, 33]. OCT2 deficient mice were protected from cisplatin-induced AKI due to the significantly impaired renal uptake of CDDP while exacerbated CDDP-caused nephrotoxicity, observed in MATE1 knockout animals, was associated with notably reduced CDDP excretion [22, 34, 35]. Additionally, gender differences in susceptibility to CDDP-induced AKI and greater intensity of CDDP-caused nephrotoxicity noticed in male rats, could be explained by reduced OCT2 expression in PTECs of female rats [36]. In line with these findings, notably reduced CDDP-induced AKI was observed in patients with nonsynonymous single-nucleotide polymorphism (SNP) in the OCT2 gene (SLC22A2 (rs316019)) [37]. Hypomagnesiemia provokes enhanced expression of OCT2 in PTECs resulting in increased uptake of CDDP [38–41]. Findings obtained in several clinical trials demonstrated that magnesium replacement may down-regulate expression of OCT2 in PTECs resulting in attenuation of CDDP-induced nephrotoxicity [38–41]. Accordingly, magnesium supplementation (8–16 milliequivalents) is now, along with short-duration and lower-volumn hydration, used for prevention of CDDP-caused renal injury [23].

In similar manner, cimetidine, a pharmacological inhibitor of OCT2, prevents OCT2-dependent renal uptake and toxicity of CDDP [41]. Katsuda and coworkers demonstrated that continuous intravenous infusion of cimetidine (20 μg/ml for 4 h) managed to efficiently prevent CDDP-caused nephrotoxicity without influencing anti-tumor activity of CDDP [41].

Interestingly, cimetidine-mediated inhibition of CDDP accumulation was significantly enhanced in Ctr1-downregulated PTECs, indicating synergistic effects of OCT2 and Ctr1 for renal uptake of CDDP. Despite the fact that down-regulated Ctr1 expression in PTECs significantly decreased their apoptosis and necrosis in vitro [42], the effects of Ctr1 deletion or inhibition on the development of CDDP-induced AKI has not yet been examined in vivo. Accordingly, future experimental and clinical studies should be focused in exploring Ctr1 as a molecular target for the enhancement of cimetidine-induced attenuation of CDDP-caused-nephrotoxicity. Additionally, it is important to highlight the fact that Ctr1 is localized on the basolateral side of both proximal and distal tubular epithelial cells. Accordingly, it was suggested that, in addition to its role in CDDP renal uptake, Ctr1 might be, in similar manner as MATE1, responsible for CDDP excretion [43]. In line with these findings, it was recently reported by Chang and coworkers that SNPs in SLC31A1/Ctr1 and SLC47A1/MATE1 genes were associated with increased urinary excretion of well-known AKI biomarkers: kidney injury molecule-1 (KIM-1), calbindin, trefoil factor 3 (TFF3), cystatin C and clusterin.

Extracellular biotransformation of CDDP begins immediately after transportation of CDDP to the apical surface of renal epithelial cells. The initial step of CDDP-induced nephrotoxicity is formation of glutathione conjugates in circulation, mediated by glutathione-S-transferase. After entering the kidney, glutathione-conjugates are cleaved to cysteinyl-glycine-conjugates by gamma glutamyl transpeptidase (GGT), which is expressed on the surface of PTECs. Aminopeptidase N (APN) converts cysteinyl-glycine-conjugates into cysteine-conjugates which are, after entering into the PTECs, further metabolized to highly reactive and nephrotoxic thiols by enzymic activity of cysteine-S-conjugate beta-lyase (CCBL) [22, 44]. Having in mind that, among all tissues, GGT has the highest activity in the kidneys, particularly on the apical surface of PTECs, this enzyme was considered as a potential target for the attenuation of CDDP-induced nephrotoxicity. However, obtained results are controversial, suggesting that enhanced GGT activity may either increase or decrease sensitivity of PTECs to CDDP [29]. More than two decades ago, Hanigan and colleagues demonstrated amelioration of CDDP-induced AKI in rats pre-treated with acivicin, a non-competitive inhibitor of GGT [45]. Additionally, the same research group showed that CDDP-induced nephrotoxicity was dependent on GGT activity and was not associated with CDDP renal uptake [46]. GGT knockout animals did not develop CDDP-induced AKI although there were no differences in CDDP accumulation between PTECs of wild-type (WT) and GGT-deficient mice [46]. Although findings obtained by Hanigan and colleagues strongly indicated an important role of GGT in toxification of CDDP, several other research groups showed opposite results suggesting that GGT could be considered as the main CDDP detoxification enzyme in the kidneys [47, 48]. Daubeuf and colleagues and Paolicchi and coworkers demonstrated that GGT products (cysteinyl-glycine-conjugates) and the GGT substrate (glutathione) were able to covalently attach to CDDP rendering it non-toxic [47, 48]. Taken together, these, on first sight opposite findings, suggest that GGT-mediated detoxification of CDDP was dependent on the complex interactions between CDDP-derived metabolites and PTEC-expressing enzymes. Indeed, GGT is, along with APN and CCBL, a member of multi-enzyme pathway which capacity to convert xenobiotic-glutathione conjugates to nephrotoxic metabolites is dependent on the synergistic activity of these three enzymes [49]. In line with these observations, Hauscheer and colleagues proposed that BNP7787 (disodium 2,2-dithio-bis-ethane sulfonate, dimesna, Tavocept™) could be considered as effective nephroprotective agent for the prevention of CDDP-induced renal dysfunction since BNP7787-derived mesna-disulfide heteroconjugates, which contain a terminal gamma-glutamate moiety (mesna-glutathione and mesna-cysteinyl-glutamate), simultanously inhibited GGT, APN and CCBL activity, attenuated generation of highly potent and nephrotixic thiols and ameliorated CDDP-induced nephrotoxicity [49, 50]. Similarly, due to their capacity to prevent the formation of a glutathione-cisplatin-conjugates, thiol agents have been tested as nephroprotective drugs in CDDP-treated patients. Among them, Amifosten was FDA-approved for the prevention of AKI in CDDP-treated patients with non-small cell lung cancer and advanced ovarian cancer [26]. Nevertheless, renoprotective effects of Amifostine were not consistently observed in CDDP-treated patients and many severe side effects including dysfunction of vestibulocochlear, gastrointestinal and cardiovascular systems significantly limited its clinical use [25, 26]. Accordingly, several recently conducted experimental and clinical trials focused their attention in the modulation of intracellular signaling pathways which were responsible for CDDP-induced cell cycle arrest or cell death.

### Molecular mechanisms responsible for CDDP-induced cell cycle arrest or cell death

CDDP-induced nephrotoxicity is dose dependent and involves necrosis, apoptosis and necroptosis of renal cells [51–53]. In vitro studies revealed that necrotic cell death is caused by high levels of CDDP, while apoptosis is caused by lower concentrations of CDDP [51, 52]. Three apoptotic pathways (extrinsic, intrinsic (mitochondrial) and endoplasmic reticulum (ER) stress pathway) may be initiated in PTECs after CDDP treatment. CDDP activates caspase-3,-8 and − 9 and induces translocation of Bax to mitochondria resulting in cytohrome c, apoptosis-inducing factor (AIF), endonuclease G release [52–57]. Inhibition of caspase-3 and caspase-9 suppressed CDDP-induced cell death [57], while cytochrome c release was diminished in CDDP-treated Bax-deficient mice [54], suggesting the important role of both extrinsic and intrinsic apoptotic pathways in the development of CDDP-induced AKI. In addition to these two pathways, CDDP-induced apoptosis of PTECs also involves the ER-stress pathway mediated by caspase 12 and calcium-independent phospholipase A2 (ER-iPLA2) [58–60]. Inhibition of caspase 12 as well as suppression of ER-iPLA2 significantly reduced apoptotic cell death of CDDP-injured PTECs and ameliorated CDDP-caused nephrotoxicity [58–60].

In addition to necrosis and apoptosis, necroptosis is also observed in renal cells after CDDP treatment. Deletion of genes involved in necroptotic pathway (receptor-interacting protein 1 (RIP1) and mixed lineage kinase domain-like protein (MLKL)) managed to protect experimental animals against CDDP-induced AKI [61, 62] indicating that pharmacological inhibitors of these molecules could be considered as possible therapeutic agents for the attenuation of CDDP-caused nephrotoxicity.

CDDP treatment induces DNA damage, dysfunction of cytoplasmatic organelles and oxidative stress in PTECs. Once the cisplatin enters PTECs, its complex interactions with the cellular environment convert it into a positively charged electrophile that has a high affinity to DNA [63]. This results in formation of intrastrand crosslink between two adjacent guanine residues within DNA [43]. More precisely, platinum atom of CDDP forms covalent bonds with N7 position of purine bases to form 1,2- or 1,3-intrastrand crosslinks and a lower percentage of interstrand crosslinks. Formation of CDDP-induced DNA adducts prevents DNA synthesis and replication causing the cell to enter in cell-cycle arrest mode. Additionally, CDDP-caused crosslinks and disrupted DNA structure resulting in the activation of DNA repair mechanisms [43]. Among them, nucleotide excision repair (NER) pathway is mainly responsible for the repair of the CDDP-induced intrastrand adducts while base excision repair (BER), homologous recombination (HR), and Fanconi anemia pathways are involved in the repair of CDDP-caused interstrand cross-links [64]. Although an increased activity of these DNA repair pathways has been associated with alleviation of CDDP-induced AKI, member proteins of these signaling cascades are not considered as ideal molecular targets for prevention of CDDP-caused nephrotoxicity since malignant cells also used enhanced activity of NER, BER and HR systems for resistance to CDDP [65].

**Fig. 1:**
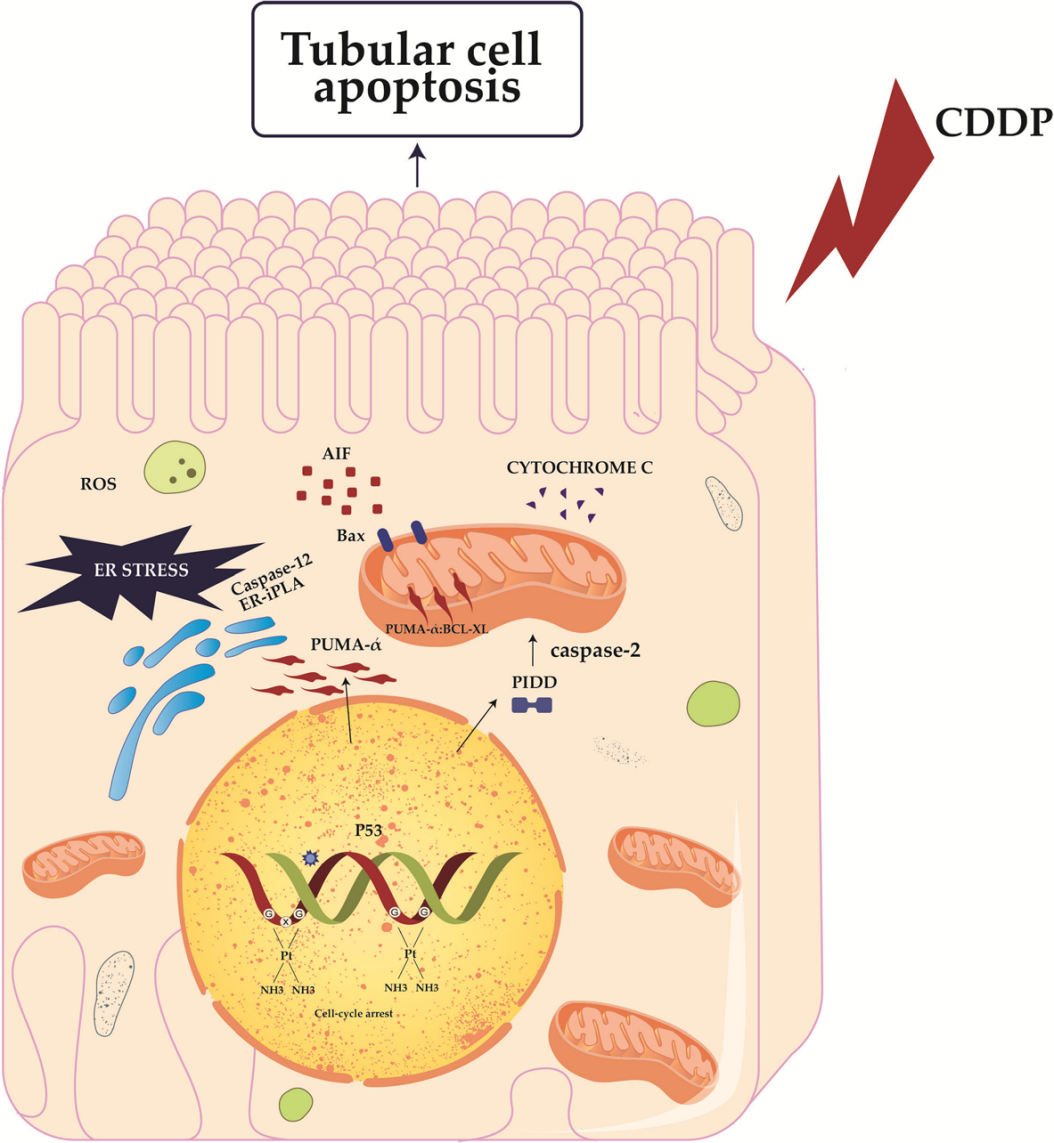
P53 signaling pathways leading to tubular cell apoptosis after cisplatin treatment. By transcriptional regulation, nuclear p53 may activate proapoptotic genes, such as PUMA-α, caspases, PIDD, and ER-iPLA2, may suppress antiapoptotic genes, including p21 and TauT. In the absence of transcription, p53 may induce apoptosis via interactions with Bcl-2 family proteins in mitochondria and/or cytosol. *Abbreviations*: Bcl-2: B-cell lymphoma 2; Bcl-xL: B-cell lymphoma-extra large; Bax: Bcl-2-associated X protein; Bak: Bcl-2 homologous antagonist killer; PUMA-α: p53 upregulated modulator of apoptosis; PIDD: p53-induced protein with a death domain; ER-iPLA2: Ca2^+^-independent phospholipase A2; Cdk2: Cyclin-dependent kinase complex; TauT: taurine transporter

Among cellular organelles, ER and mitochondria are the most severe injured by CDDP (Fig. 1) [43]. CDDP-caused alterations in translation lead to the accumulation of misfolded proteins within ER, resulting in the development of ER stress. At the same time, positively charged CDDP electrophile preferentially accumulate in the negatively charged mitochondria affecting their function [66]. Mitochondrial dysfunction and consequent reduced ATP synthesis force CDDP-injured PTECs to function in a starvation mode. Thus, CDDP-induced prolonged ER stress and hypoxic injury provoke caspase-mediated apoptosis or induce extensive production of free radicals and reactive oxygen species (ROS) resulting in the development of oxidative stress [43]. In addition to mitochondrial dysfunction, CDDP may provoke oxidative stress in PTECs by inducing ROS formation in the microsomes through the activation of cytochrome P450 system [20, 67]. Administration of antioxidants (vitamins C, E, selenium, alpha lipoic acid, dimethylthiourea (DMTU)) showed beneficial, renoprotective effects against CDDP-caused nephrotoxicity [68–71], confirming the important pathogenic role of oxidative stress in the development of CDDP-induced AKI. Since DMTU significantly suppressed p53 activation in CDDP-injured cells, p53 was considered as important downstream target of CDDP-generated ROS.

Several lines of evidence confirmed that P53 protein was critically involved in the development of CDDP-induced nephrotoxicity [72]. CDDP treatment provokes increased expression and activation of p53 in injured kidneys where p53 regulates apoptosis of PTECs through transcriptional activation and repression of genes whose promoters contain p53-binding sites (Fig. 1) [72]. CDDP-caused alterations in DNA structure activates molecular sensors for DNA damage: ataxia telangiectasia (ATM) and Rad3-related (ATR) proteins, which activate Checkpoint kinase 2 (Chk2), resulting in phosphorylation and activation of p53 [65, 72]. P53-up-regulated modulator of apoptosis (PUMA)-훼, p53-induced protein with death domain (PIDD), caspases 6 and 7, p21 protein and Taurine transporter (TauT) have the most prominent role in p53-dependent modulation of CDDP-induced acute renal failure. Activated p53 protein induces increased accumulation of PUMA-훼 in mitochondria of CDDP-injured tubular cells where this protein interacts with Bcl-xL. An enhanced PUMA-훼:Bcl-xL crosstalk enables Bax-mediated permeabilization of mitochondrial membrane and consequent release of cytochrome c, resulting in caspase activation and apoptosis of renal tubular cells [73]. Accordingly, reduced induction of PUMA-훼 was observed in p53 deficient animals which were protected against CDDP-induced nephrotoxicity [73], indicating the importance of p53-dependent activation of PUMA-훼 for apoptosis of PTECs. In similar manner, p53 promotes translation of PIDD which, through the activation of caspase 2, induces mitochondrial release of AIF leading to the chromatin condensation and programmed cell death of CDDP-injured PTECs [55]. In vitro and in vivo studies revealed important role of p53 for caspase 6 and 7-dependent apoptosis of CDDP-injured PTECs. Pharmacological inhibition as well as genetic deletion of p53 blocked the activation of both executioner caspases and protected PTECs from CDDP-caused apoptosis resulting in alleviation of CDDP-induced nephrotoxicity [74].

Although induction of pro-apoptotic molecules is dominant effect of p53 activation in CDDP-injured PTECs, p53 interferes with anti-apoptotic molecules (p21 and TauT) regulating their renoprotective function, as well. P53-induces down-regulated expression of TauT gene and, consequently, increases apoptosis in CDDP-injured renal cells [75]. Similarly, p53 may regulate activity of p21 protein, a well-known anti apoptotic regulator of cell survival [72]. Induction of p21 protein in renal cells is considered as an important renoprotective mechanism against CDDP-caused nephrotoxicity since both genetic deletion or pharmacological inhibition of p21 significantly augmented CDDP-provoked injury of PTECs [76, 77]. Mechanistically, p21-mediated inhibition of cyclin-dependent kinase 2 (CDK2) was mainly responsible for p21-dependent nephroprotection [78]. Having in mind that CDDP-induced toxicity depends on CDK2 activity, and that CDK2 inhibition protected kidney cells from CDDP-induced cell death [79, 80], p21-dependent suppression of CDK2 could be used as potentially useful therapeutic approach for attenuation of CDDP-induced AKI.

In addition to the regulation of apoptosis, p53 may contribute to the development of CDDP-caused nephrotoxicity by modulating autophagy which, as an adaptive mechanism, promotes PTECs survival during AKI [72, 81]. Immediately after exposure of PTECs to CDDP, markers of autophagy (Beclin 1, Microtubule-associated proteins 1A/1B light chain 3B (LC3), and Autophagy-related protein 5 (Atg5)) were significantly increased in CDDP-injured renal cells, indicating development of autophagy [82]. Accordingly, inhibition of autophagy in CDDP-treated animals resulted in increased apoptotic cell death of PTECs [83]. Similarly, an increased DNA damage and an enhanced p53 activation were observed in PTECs of mice that lack autophagy related genes, confirming capacity of p53 to regulate renoprotective autophagy in CDDP-injured PTECs [82, 84, 85]. In CDDP-injured PTECs, CDDP activates AMP-activated protein kinase (AMPK), a signaling molecule that regulates autophagic protection against CDDP-induced AKI [81]. Accordingly, genetic deletion or pharmacological inhibition of AMPK resulted in repressed autophagy in CDDP-injured PTECs, followed by increased DNA damage and consequently enhanced activation of p53. Having in mind that p53 regulates autophagy by inactivating mammalian target of rapamycin (mTOR) pathway via AMPK, p53-based modulation of AMPK activity could be considered as an important mechanism for p53-dependent regulation of CDDP-induced AKI [81].

P53 regulates activation of pro-apoptotic microRNA (miR)-375 and cytoprotective miR-34a in CDDP-injured PTECs [86]. Upon CDDP administration, p53 and nuclear transcription factor-kappa B (NF-κB) collaboratively induce enhanced expression of miR-375 which suppressed activation of nephroprotective hepatocyte nuclear factor 1 homeobox B (HNF-1β), resulting in renal tubular cell apoptosis and nephrotoxicity. Additionally, pharmacological inhibition of p53 or NF-κB resulted in down-regulated expression of miR-375 which led to the alleviation of CDDP-induced AKI. In contrast to miR-375, inhibition of miR-34a induced increased apoptosis of PTECs while enhanced expression of miR-34a promoted survival of CDDP-injured PTECs, indicating an important cytoprotective role of miR-34a in CDDP-induced nephrotoxicity [87].

Based on all these findings, p53 represents potential molecular target for alleviation of CDDP-caused neprotoxicity. Experimental studies already demonstrated that temporary and reversible p53 suppression during cancer therapy can be relatively safe [88]. Additionally, selective, pharmacological inhibitors of p53 may be specifically delivered to renal cells without affecting primary tumor or metastatic lesions [89, 90]. Accordingly, p53 antagonists could protect PTECs from CDDP-caused injury without affecting CDDP-induced anti-tumor effects in malignant cells. Furthermore, in some tumors p53 protein was responsible for tumor resistance to chemotherapeutics [81]. In these patients, systemic administration of p53 antagonists may result in nephroprotection and at the same time could sensitize malignant cells to anticancer drugs promoting their therapeutic efficacy [91]. Despite these promising expectations, it should be highlighted that due to the complex role that p53 has in regulation of cell survival, nephroprotection due to the selective p53 renal inhibition should be investigated, in detail, in CDDP-treated tumor-bearing animal models before it will be considered as one of possible therapeutic approaches for CDDP-treated patients.

### Signaling pathways responsible for extensive production of inflammatory cytokines in CDDP-injured kidneys

CDDP-induced activation of NF-*κ*B, poly ADP-ribose polymerase-1 (PARP-1) and toll-like receptors (TLRs) pathways in PTECs and renal-infiltrated immune cells results in extensive production of inflammatory cytokines [92]. CDDP induces the phosphorylation and subsequent translocation of NF-κB to the nucleus, where activated NF-κB promotes transcription of tumor necrosis factor alpha (TNF-α) and other inflammatory cytokines (IL-1, IL-6, IL-18) [93]. Similarly, CDDP-induced DNA damage results in activation of PARP-1 which promotes apoptosis of CDDP-injured PTECs or induces up-regulation of TNF-alpha, IL-1 and IL-6 genes contributing to the development of AKI [94]. Genetic deletion of PARP-1 completely diminished CDDP-caused renal injury and inflammation, while administration of selective, pharmacological inhibitor of PARP-1 (PJ-34) efficiently protected against CDDP-induced nephrotoxicity [94, 95].

Several lines of evidence demonstrated that activation of TLR-4, TLR-2 and TLR-9 may modulate CDDP-induced acute renal injury and inflammation [96, 97]. Animals deficient in TLR-4 showed lower serum levels of TNF-α and were protected from CDDP induced renal toxicity [98]. Activation of TLR-4 on renal parenchymal cells activates p38 mitogen-activated protein kinase (MAPK) pathway, increased production of TNF-α and led to the development of inflammation in CDDP-injured kidneys [99]. Several damage associated molecular patterns (DAMPs): heat shock proteins (HSP)-60,-70, β-defensin-2, gp96 and HMGB1, released from CDDP-injured PTECs, were designated as possible endogenous TLR-4 ligands capable to initiate immune response in TLR-4:TNF-α-dependent manner [99]. Additionally, CDDP-induced extensive release of endogenous TLR-4 ligands may activate inflammasome complex in renal infiltrated immune cells resulting in enhanced production of inflammatory cytokines (IL-1 and IL-18), further contributing to the development of renal inflammation [100]. In line with these findings, future experimental and clinical studies should explore HMGB1, β-defensin-2, gp96, HSP-60 and 70 as potential molecular targets for the TLR-4-related attenuation of CDDP-caused AKI.

In addition to the activation of TLR-4 pathway, CDDP treatment affects TLR-2 and TLR-9 signaling, as well. Andrade-Silva and colleagues recently demonstrated that TLR-2, opposite to TLR-4, protects against CDDP-induced nephrotoxicity by promoting development of autophagy in CDDP-injured renal cells [96]. Genetic deletion of TLR-2 down-regulated expression of autophagy-related genes in PTECs which resulted in severe exacerbation of renal dysfunction and increased mortality rate of CDDP-treated TLR-2 knockout animals [96]. Similarly as TLR-2, TLR-9 also plays renoprotective role in CDDP-caused nephrotoxicity. As recently demonstrated by Alikhan and colleagues, presence of immunosuppressive T regulatory cells (Tregs) in CDDP-injured kidneys is, at least partially, regulated by TLR-9 [101]. TLR-9 deficient mice had significantly reduced number of Tregs in injured kidneys and consequently developed enhanced immune response and inflammation in CDDP-injured kidneys [101]. Since TLR-9 responds to mitochondrial DAMPs [102], TLR-9-dependent expansion of renal-infiltrated Tregs was probably elicited as a cytoprotective mechanism against CDDP-induced mitochondrial damage. In line with these findings, specific delivery of TLR-2 and TLR-9 agonists in CDDP-injured PTECs and consequent induction of autophagy and expansion of Tregs should be tested in future experimental studies as new therapeutic approach against CDDP-induced AKI.

### Cytokine networking in CDDP-injured kidneys: a potential target for renoprotection

**Fig. 2:**
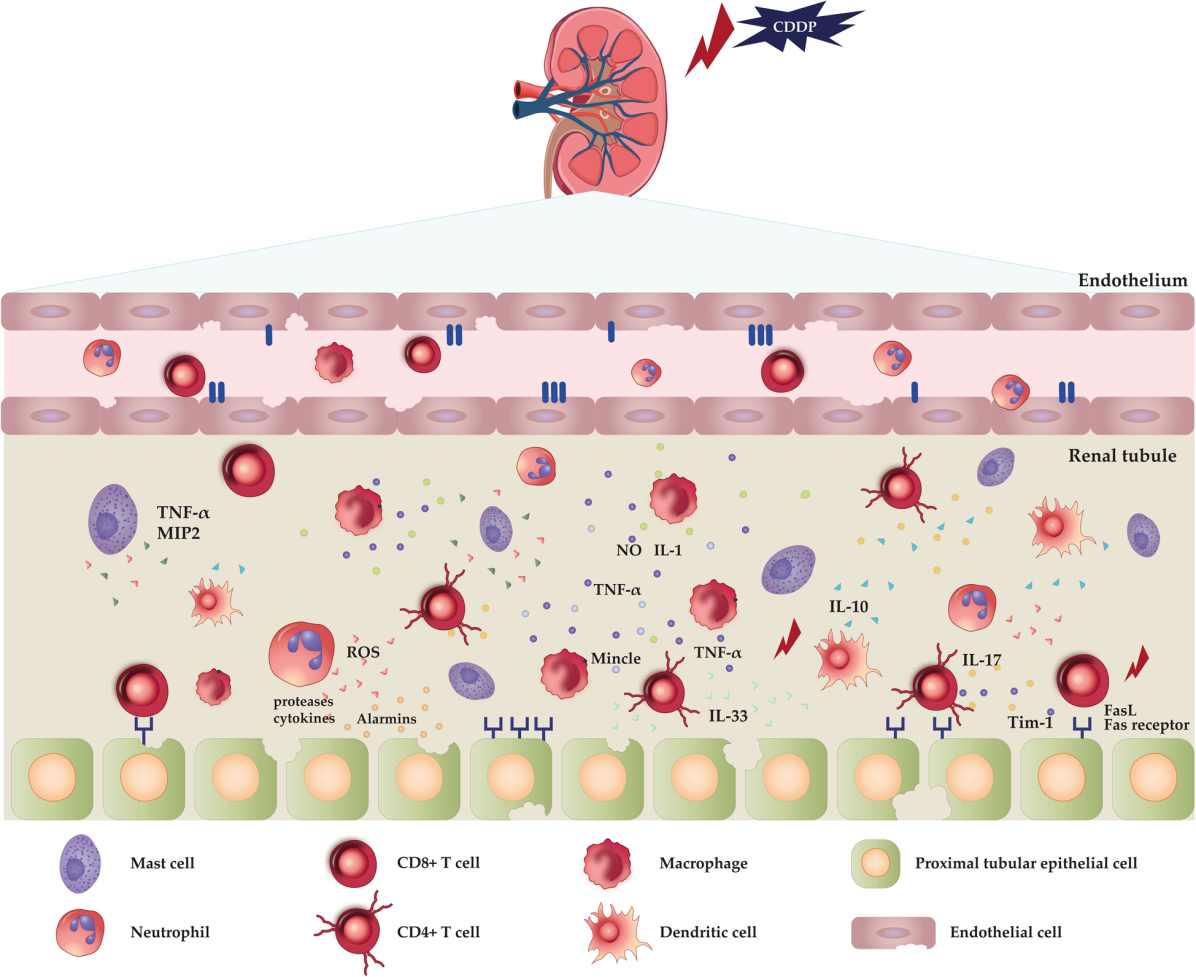
Cell subtypes that play crucial role in the pathogenesis of cisplatin-induced AKI. Cisplatin-induced AKI involves the coordinated actions of proximal tubular epithelial, endothelial, innate and adaptive immune cells. *Abbreviations*: ROS: Reactive oxygen species; IL: Interleukin; TNF-α: Tumor necrosis factor alpha; MIF: Macrophage migration inhibitory factor; Mincle: Macrophage-inducible C-type lectin; CXCL1: Chemokine (C-X-C motif) ligand 1; Kim-1: Kidney injury molecule-1; ICAM-1: Intercellular adhesion molecule-1

Development and progression of renal inflammation upon CDDP treatment is controlled and regulated by complex interaction between inflammatory and immunosuppressive cytokines produced either by CDDP-injured PTECs or renal-infiltrated immune cells (Fig. 2). Among inflammatory cytokines, TNF-α appears to play a central role in the inflammatory response triggered by CDDP [103]. CDDP increases both serum and urine concentrations of TNF-α [103, 104], while CDDP-induced nephrotoxicity was attenuated in TNF-α-deficient mice as well as in mice treated with TNF-α inhibitors (pentoxifylline) or TNF-α neutralizing antibodies [103]. Renal parenchymal cells (mesangial cells, glomerular cells, endothelial and renal tubular cells), macrophages and CD4 + T helper lymphocytes are cellular sources of TNF-α in CDDP-induced AKI [104]. Depletion of T cells reduced TNF-α production and protected against CDDP-induced AKI, suggesting crucial role of T cells in TNF-α-driven renal inflammation elicited by CDDP [104, 105]. Major role of TNF-α is to stimulate the production of other inflammatory cytokines and chemokines and to promote recruitment of inflammatory cells in CDDP-injured kidneys [93, 106–108]. Significant increase in mRNA levels of macrophage inflammatory protein-2 (MIP-2), monocyte chemoattractant protein-1 (MCP-1), IL-1β, and TGF-β has been observed in the kidneys of CDDP-treated mice [109]. MIP-2, MCP-1 and IL-1β are involved in recruitment of circulating monocytes in the inflamed renal parenchyma [110] while TGF-β has crucially important role in the development of renal fibrosis [111]. Accordingly, an enhanced production of these inflammatory mediators, further promoted TNF-α-driven AKI triggered by CDDP. Importantly, production of MIP-2, MCP-1, IL-1β and TGF-β in CDDP-injured kidneys was TNF-α-dependent [109]. Selective inhibition of TNF-α significantly reduced production of MIP-2, MCP-1, IL-1β, TGF-β and attenuated AKI in CDDP-treated animals [109].

TNF-α induces expression of adhesion molecules on renal endothelial cells and promotes influx of circulating leucocytes in the inflamed renal parenchyma [112, 113]. Among selectins and integrins, intercellular adhesion molecule-1 (ICAM-1) has shown to be the most important for TNF-α-driven migration of immune cells into CDDP-injured kidneys [114]. Accordingly, reduced infiltration of immune cells, accompanied with attenuated inflammation and reduced PTECs damage correlated with down-regulated expression of ICAM-1 and TNF receptors (TNFR1–2) in CDDP-injured kidneys [115]. Although TNFR1 was responsible for TNF-α-induced systemic and anti-tumor effects [116], several lines of evidence demonstrated that TNFR2 rather than TNFR1 mediates cytotoxic and inflammatory actions of TNF-α in CDDP-injured kidneys [116, 117]. Compared to TNFR1 knockout mice, TNFR2 deficient animals showed reduced serum and kidney levels of TNF-α and were less susceptible to CDDP-induced TNF-α-driven renal failure. Accordingly, TNFR2 blockade should be evaluated in future experimental studies as potentially new therapeutic approach that might reduce nephrotoxicity without affecting systemic and anti-tumor effects of CDDP-induced TNF-α.

An increased concentration of TNF-α in CDDP-injured kidneys was usually accompanied with significant elevation of IL-8, IL-1β, and IL-18 in renal parenchyma of CDDP-treated animals [118]. High renal concentration of these inflammatory cytokines further promoted TNF-α-driven inflammation and CDDP-induced renal failure [118]. Several lines of evidence suggest that IL-8, in similar manner as TNF-α, regulates ICAM-1-dependent influx of circulating leukocytes in injured kidneys [119]. Administration of α-melanocyte-stimulating hormone analogue, which inhibited IL-8-dependent expression of ICAM-1, significantly reduced recruitment of inflammatory cells in the kidneys and remarkably attenuated AKI [120].

Having in mind that IL-1β and IL-18 are synthesized in inactive preforms and become activated in a caspase-1 dependent manner [121, 122], caspase-1 was designated as a potential molecular target for attenuation of CDDP-induced nephrotoxicity. Faubel and coworkers revealed that caspase-1 activity was remarkably increased in the kidneys of CDDP-treated animals and that renal dysfunction was significantly reduced in caspase-1 deficient mice [123]. Moreover, genetic deletion of caspase-1 significantly attenuated renal concentration of IL-1β and IL-18 and reduced influx of circulating neutrophils in CDDP-injured kidneys [123]. Interestingly, selective inhibition of IL-1β and IL-18 did not alleviate CDDP-induced inflammation, suggesting that other caspase-1-processed cytokines, also contributed to the progression of CDDP-caused AKI [118].

IL-1α, like IL-1β, is a pro-inflammatory cytokine which initiates the same biological processes as IL-1β [124]. In line with these findings, Lee and colleagues revealed an important pathogenic role of IL-1α in the pathogenesis of CDDP-induced nephrotoxicity and suggested that this cytokine may be activated by caspase-1 during the progression of CDDP-caused inflammation [125]. Compared with vehicle-treated mice, concentration of renal IL-1α was significantly increased in CDDP-treated animals. Importantly, genetic deletion of IL-1α efficiently protected against CDDP-induced AKI and the extent of CDDP-caused renal injury was similar as it was observed in caspase-1 deficient animals [125].

In addition to TNF-α, IL-1α-β, IL-8 and IL-18, recently published studies indicated important role of IL-17A and IL-33 in pathogenesis of CDDP-induced AKI [126, 127]. An increased expression of IL-17A was observed in CDDP-injured kidneys [127]. CDDP treatment induces activation of inflammasome complex in renal infiltrated leukocytes, resulting in extensive IL-17A production. Innate immune cells (neutrophils and natural killer (NK) cells) were designated as the main IL-17A producing cells in CDDP-induced nephrotoxicity while IL-17A-producing T cells were not involved in CDDP-caused renal inflammation since detrimental effects of IL-17A on renal structure and function were also observed in RAR-related orphan receptor gamma T (RORγT) deficient mice that lack effector CD4 + Th17 lymphocytes [127]. Administration of anti-IL-17A antibody efficiently protected against CDDP-induced nephrotoxicity, confirming important pathogenic role of IL-17A in CDDP-caused AKI [127].

IL-33 has the capacity to alter immune response elicited in CDDP-injured kidneys [128]. Akcay and colleagues showed that IL-33 promotes inflammation in CDDP-injured kidneys by acting as an alarmin [126]. IL-33, released from CDDP-injured PTECs, binds to IL-33 receptor (ST2) on renal-infiltrated CD4+ T cells and promotes secretion of inflammatory cytokines and chemokines (particularly TNF-α and neutrophil chemoattractant CXCL1), contributing to the development of acute renal inflammation [126]. In line with these observations, increased concentration of IL-33 was noticed in sera and kidneys of CDDP-treated animals [126]. Importantly, notably reduced acute tubular necrosis and apoptosis was observed in mice treated with a soluble IL-33 receptor (sST2), while administration of recombinant IL-33 (rIL-33) exacerbated CDDP-induced AKI [126], indicating pro-inflammatory role of IL-33 in the pathogenesis of CDDP-caused nephrotoxicity. Opposite to these findings are results recently obtained by Stremska and colleagues who demonstrated that two subpopulations of renal-infiltrated immunosuppressive and renoprotective cells (Tregs and innate lymphoid cells, ILC) expressed ST2 receptor and extensively proliferated in the presence of IL-2 and IL-33 [128]. In line with these observations, Stremska and co-workers designed IL-233, a novel IL-2 and IL-33 hybrid cytokine, whose administration efficiently expanded ST2 expressing Tregs and ILCs in CDDP-injured kidneys and completely attenuated AKI and inflammation [128]. These results strongly suggest nephroprotective potential of IL-233 against CDDP-caused AKI which should be confirmed in future clinical trials.

Inflammatory cytokines and chemokines are extensively produced in early phases of CDDP-induced AKI [129]. Several lines of evidence suggested that elevation in urine concentration of pro-inflammatory cytokines and chemokines may be considered as an important parameter for early diagnosis of CDDP-caused nephrotoxicity. Increased levels of interferon γ-induced protein-10 (IP-10), keratinocyte chemoattractant (KC) and granulocyte-colony stimulating factor (G-CSF) were detectable in the urine of CDDP-treated mice as early as 6 hours after treatment, long before the serum creatinine or urea nitrogen levels were increased [22, 129]. Likewise, increased urinary levels of KC, IL-2, MCP-1, GM-CSF and IL-8 were noticed in initial phase of on-going renal inflammation in CDDP-treated dogs and their concentration correlated with the progression of renal dysfunction [130]. Accordingly, measurement of urine levels of inflammatory mediators should be considered as an important approach for early diagnosis and prevention of CDDP-induced nephrotoxicity [22].

Among anti-inflammatory and immunosuppressive cytokines, it was well documented that IL-10, produced mainly by renal-infiltrated Tregs and tolerogenic dendritic cells, efficiently reduced CDDP-induced AKI and associated inflammation [131–133]. Soon after CDDP administration, IL-10 producing Tregs migrated into injured kidneys and suppressed detrimental TNF-α, IL-1 and IL-17-driven immune response [134]. Antibody-mediated depletion of endogenous Tregs leads to exacerbation of CDDP-induced AKI, while their transfer protected against CDDP-induced nephrotoxicity [134]. Adoptive transfer of Tregs notably reduced production of inflammatory cytokines (TNF-α and IL-1) in renal macrophages, suppressed activation of IL-17-producing neutrophils and NK cells, significantly attenuated inflammation and completely restored renal function of CDDP-treated animals [134]. In similar manner as Tregs, IL-10 producing tolerogenic dendritic cells (DCs) have a protective role in CDDP-induced AKI. DC-derived IL-10 inhibits production of inflammatory cytokines in renal infiltrating T cells and macrophages [114, 135–137]. Depletion of IL-10 producing DCs significantly exacerbated CDDP-induced nephrotoxicity while their passive transfer restored renal function in CDDP-treated animals [135, 136]. In line with these findings, cell-based therapy based on autologous transplantation of IL-10 producing Tregs and tolerogenic DCs in CDDP-injured kidneys should be further explored as potentially new therapeutic approach for renoprotection of CDDP-treated patients.

In addition to IL-10, IL-6 had been also considered as an important anti-inflammatory cytokine which may protect against CDDP-induced nephrotoxicity [138]. Administration of CDDP provoked increased production of IL-6 in injured kidneys. An enhanced expression of IL-6 resulted in up-regulation of anti-oxidative enzymes in inflamed renal parenchyma which prevented the development of CDDP-caused renal dysfunction. In an analogy, genetic deletion of IL-6 significantly reduced activity of superoxide dismutase and increased expression of oxidative stress markers in CDDP-injured kidneys [138].

### The impact of renal-infiltrated immune cells on the development and progression of CDDP-induced AKI

CDDP treatment cause morphological and/or functional changes in tubular and endothelial cells which leads to an influx of mast cells, neutrophils, macrophages, NK cells and T lymphocytes into the injured kidneys where these immune cells play aggressive or protective role (Fig. 2).

Mast cells play an important pathogenic role in CDDP-induced AKI. Selective depletion of mast cells attenuated renal injury caused by CDDP, reduced recruitment of leukocytes to the injured kidneys and notably down-regulated serum levels of TNF-α, suggesting that mast cells mainly mediated CDDP-induced AKI in TNF-α-dependent manner [139]. Mast cell-derived TNF-α and macrophage inflammatory protein 2 (MIP-2) promote recruitment of neutrophils in CDDP-injured kidneys significantly contributing to the aggravation of on-going inflammation [140].

An increase in total number of renal infiltrated neutrophils correlates with the extent of CDDP-induced AKI [141, 142]. Activated neutrophils, through the release of ROS, proteases, and inflammatory cytokines cause tubular damage resulting in extensive release of DAMPs and alarmins [143]. Significant reduction of renal-infiltrated neutrophils can be achieved by TNF-α inhibitors, TLR-4 antagonists or anti-ICAM-1 antibodies [99, 115, 133, 144]. Nevertheless, depletion of neutrophils was not enough to completely protect from CDDP-induced nephrotoxicity [135, 141], suggesting that renal-infiltrating neutrophils are not the only effector immune cells in the pathogenesis of AKI caused by CDDP.

Macrophages play an important pathogenic role in CDDP-induced nephrotoxicity [145, 146]. An increased number of macrophages was observed in injured kidneys 2 days after CDDP administration. CDDP induces activation of inflammasome, p38 MAPK and NF-kB pathways in renal macrophages resulting in increased production of superoxide anions, nitric oxide (NO), IL-1 and TNF-α [106, 145]. It was recently revealed that macrophage-inducible C-type lectin (Mincle), transmembrane pattern recognition receptor, is selectively expressed in renal infiltrating M1 macrophages, and is responsible for generation and maintenance of inflammatory phenotype of these cells during CDDP induced AKI [146]. Mincle expression is regulated by TLR-4/NF-kB signaling pathway and its down-regulation resulted in generation of nephroprotective, alternatively activated macrophages whose adoptive transfer efficiently protected against CDDP-induced nephrotoxicity [146]. In line with these findings, Mincle represents a potential cellular target for alternative activation of renal macrophages in CDDP-treated patients and its nephroprotective potential should be explored in future experimental and clinical studies.

CD4+ T cell deficient and, to a lesser extent, CD8-deficient mice were less susceptible to CDDP-induced nephrotoxicity compared with WT animals, demonstrating importance of T lymphocytes in the pathogenesis of CDDP-induced nephrotoxicity [104]. While CD4+ T helper cells in paracrine manner (through the production of TNF-α, IL-17, IL-33 and IL-10) orchestrate immune response in CDDP-injured kidneys, cytotoxic CD8+ T lymphocytes in juxtacrine, contact dependent manner, induce damage of renal cells. CDDP treatment increases expression of Fas receptors on renal tubular cells enabling apoptosis of these cells due to their interaction with FasL expressing renal infiltrating CD8+ T lymphocytes [147]. CDDP induces enhanced expression of T cell immunoglobulin mucin 1 (Tim-1) on PTECs [148]. Tim 1 acts as a costimulatory molecule playing important role in activation of renal-infiltrated T cells [148]. Administration of Tim-1-blocking antibody inhibited activation of renal-infiltrated CD4+ helper and CD8+ cytotoxic T cells, reduced apoptosis of PTECs and protected against CDDP-induced AKI, indicating therapeutic and nephroprotective potential of Tim-1 that should be further explored in up-coming experimental and clinical studies [148].

## Conclusions

During the last decade, a large number of experimental studies have shed new light on molecular and cellular mechanisms of CDDP-induced nephrotoxicity. Signaling pathways which regulate cell survival, metabolism and immune response are affected by CDDP. However, most of these pathways are, at the same time, crucially involved in cytotoxic activity of CDDP against tumor cells and potential alterations in their function might mitigate CDDP-induced anti-tumor effects. Accordingly, despite the fact that many molecules were designated as potential therapeutic targets for renoprotection against CDDP, modulation of CDDP-induced nephrotoxicity is still a balance on the knife edge between renoprotection and tumor toxicity. Design of new renoprotective strategies that would not limit CDDP-induced tumoricidal effects should rely on the identification of the structural and functional differences between CDDP-injured renal and tumor cells. Their implementation would open new avenues in chemotherapy significantly enhancing clinical utility of CDDP.

## Abbreviations

AIF: Apoptosis-inducing factor
AKI: Acute kidney injury
AMPK: AMP-activated protein kinase
APN: Aminopeptidase N
Atg5: Autophagy-related protein-5
ATM: ataxia telangiectasia
ATP: Adenosine triphosphate
ATR: Rad3-related protein
Bax: Bcl-2-associated X protein
Bcl-xL: B-cell lymphoma-extra large
BER: base excision repair
BNP7787: disodium 2,2-dithio-bis-ethane sulfonate, dimesna, Tavocept™
CCBL: cysteine-S-conjugate beta-lyase
CD: Cluster of differentiation
CDDP: Cisplatin
CDK2: Cyclin-dependent kinase complex
Chk2: Checkpoint kinase 2
Ctr: Copper transporter
CXCL1: Chemokine (C-X-C motif) ligand 1
DAMPs: Damage associated molecular patterns
DCs: Dendritic cells
DMTU: dimethylthiourea
DNA: Deoxyribonucleic acid
ER: Endoplasmic reticulum
ER-iPLA2: Ca2^+^-independent phospholipase A2
FasL: Fas ligand
FDA: Food and Drug Administration
GGT: Gamma glutamyl transpeptidase
HMGB1: High mobility group box 1 protein
HNF-1β: hepatocyte nuclear factor 1 homeobox B
HR: homologous recombination
HSP: Heatschock protein
ICAM-1: Intercellular adhesion molecule-1
IL: Interleukin
ILC: innate lymphoid cells
KIM-1: kidney injury molecule-1
LC3: Microtubule-associated proteins 1A/1B light chain 3B
MAPK: Mitogen-activated protein kinase
MATE1: Multidrug extrusion transporter 1
Mincle: Macrophage-inducible C-type lectin
MIP-2: Macrophage inflammatory protein 2
miR: microRNA
MLKL: Mixed lineage kinase domain-like protein
mTOR: mammalian target of rapamycin
NER: Nucleotide excision repair
NFkB: Nuclear factor kappa B
NK cells: Natural killer cells
NO: Nitric oxide.
OCT2: Organic cation transporter 2
PARP-1: poly ADP-ribose polymerase-1
PIDD: p53-induced protein with a death domain
PTECs: proximal tubular epithelial cells
PUMA-α: p53 upregulated modulator of apoptosis
rIL-33: Recombinant interleukin-33
RIP1: Receptor-interacting protein 1
RORγT: RAR-related orphan receptor gamma T
ROS: Reactive oxygen species
SLC: Solute carrier family
SNP: single-nucleotide polymorphism
TauT: Taurine transporter
TFF3: trefoil factor 3
Tim-1: T cell immunoglobulin mucin 1
TLR: Toll like receptor
TNFR: Tumor necrosis factor receptor
TNF-α: Tumor necrosis factor alpha
Tregs: Regulatory T cells
WR2721: ethanethiol, 2-[(3-aminopropyl)amino] dihydrogen phosphate ester
WT: Wild type
